# Advanced papillary carcinoma of the breast presenting as an ulcerated anterior chest wall tumour: case report

**DOI:** 10.1186/1749-8090-8-S1-O248

**Published:** 2013-09-11

**Authors:** U Abubakar, JN Legbo, SM Sahabi, AC Opara, IR Jamalu

**Affiliations:** 1 Cardiothoracic Surgery Unit, Department of Surgery, Usmanu Danfodiyo University Teaching Hospital, Sokoto, Nigeria; 2 Department of Surgery, Usmanu Danfodiyo University Teaching Hospital, Sokoto, Nigeria; 3 Histopathology Department, Usmanu Danfodiyo University Teaching Hospital, Sokoto, Nigeria

## 

Papillary carcinoma of the breast is a rare malignant tumor accounting for 1-2% of all breast cancers in women. Papillary carcinomas of the chest wall are always secondary from thyroid, thymus and ovaries. Other variants of breast cancer metastasizing to the chest wall have been reported. We report a 67 year old woman presented to us with an ulcerated anterior chest wall mass of 1 year duration, bilateral axillary, supraclavicular and cervical swellings of 8 months duration. There is history of breast lump which was noticed 5 years ago. No history of breast malignancy in the past and no family history of breast cancer. Examination revealed an ulcerated, nodular mass over the sternal angle which measured 14cmX12cmX4cm; she had bilateral axillary, supraclavicular and cervical lymphadenopathy which were non-tender and matted. She had a firm, non-tender right breast lump measuring 6cmX4cm. Chest examination and abdominal examinations were essentially normal. Chest X-ray revealed erosion of sternal bone without any evidence of intrathoracic extension (see fig). Abdominal ultrasound and thyroid scans were normal. Histology of the mass revealed papillary carcinoma primary. Biopsy of the right breast also revealed papillary carcinoma. She had excision of the fungating tumor and primary closure of defect. She subsequently had combination chemotherapy using ondasetron, cyclophosphamide, adriamycin and cisplantin. Papillary carcinoma of the breast is rare and rarely metastasizes to the chest wall. The diagnosis metastatic chest wall tumor requires meticulous history taking, clinical examination and relevant investigations to ascertain the primary origin of the carcinoma and also the extent of spread of the carcinoma.

